# Generalized laws of refraction and reflection at interfaces between different photonic artificial gauge fields

**DOI:** 10.1038/s41377-020-00411-7

**Published:** 2020-12-22

**Authors:** Moshe-Ishay Cohen, Christina Jörg, Yaakov Lumer, Yonatan Plotnik, Erik H. Waller, Julian Schulz, Georg von Freymann, Mordechai Segev

**Affiliations:** 1 Physics Department, Technion—Israel Institute of Technology, Haifa, 32000 Israel; 2 Solid State Institute, Technion—Israel Institute of Technology, Haifa, 32000 Israel; 3 Physics Department and Research Center OPTIMAS, TU Kaiserslautern, 67663 Kaiserslautern, Germany; 4 Department of Physics, The Pennsylvania State University, State College, PA 16802 USA; 5 Fraunhofer Institute for Industrial Mathematics ITWM, 67663 Kaiserslautern, Germany

**Keywords:** Optical physics, Optical physics

## Abstract

Artificial gauge fields the control over the dynamics of uncharged particles by engineering the potential landscape such that the particles behave as if effective external fields are acting on them. Recent years have witnessed a growing interest in artificial gauge fields generated either by the geometry or by time-dependent modulation, as they have been enablers of topological phenomena and synthetic dimensions in many physical settings, e.g., photonics, cold atoms, and acoustic waves. Here, we formulate and experimentally demonstrate the generalized laws of refraction and reflection at an interface between two regions with different artificial gauge fields. We use the symmetries in the system to obtain the generalized Snell law for such a gauge interface and solve for reflection and transmission. We identify total internal reflection (TIR) and complete transmission and demonstrate the concept in experiments. In addition, we calculate the artificial magnetic flux at the interface of two regions with different artificial gauge fields and present a method to concatenate several gauge interfaces. As an example, we propose a scheme to make a gauge imaging system—a device that can reconstruct (image) the shape of an arbitrary wavepacket launched from a certain position to a predesigned location.

## Photonics: Manipulating light with artificial fields

Artificial gauge fields are a technique to engineer the potential landscape such that neutral particles mimic the dynamics of charged particles driven by external fields. Researchers in Israel and Germany, led by Mordechai Segev at Technion Israel Institute of Technology, and Georg von Freymann from the University of Kaiserslautern, Germany, and their students Moshe-Ishay Cohen and Christina Joerg, studied theoretically and experimentally what happens when waves are incident at the interface between two photonic systems made from the same material, with the only thing making them different being their artificial gauge fields. The team formulated the generalized laws of refraction and reflection at such “gauge interfaces”, and demonstrated the concepts with micro-printed waveguides arrays with different tilt angles. The research demonstrates that several interfaces between regions with differing gauge fields could be used to develop novel photonic devices.

## Introduction

Snell’s law and the Fresnel coefficients are the cornerstones of describing the evolution of electromagnetic waves at an interface between two different media. By cascading several such systems, each with its own optical properties, it is possible to design complex structures that give rise to various important devices and systems, such as lenses, waveguides^1^, resonators, photonic crystals^2^, and even localization phenomena, when random interfaces are involved^3^. The behavior of waves in the presence of an interface can exhibit fundamental features, e.g., total internal reflection (TIR), back-refraction for negative-positive refraction index interfaces^4,5^, and even confinement of states to the interface itself, such as Tamm and Shockley states^6,7^, plasmon polaritons^8,9^, Dyakonov states^10,11^ and topological edge states^12–14^. Traditionally, the Fresnel equations describe the reflection and transmission of electromagnetic waves at an interface separating two media with different optical properties. These can be two materials with different permittivities or two different periodic systems (photonic crystals) composed of the same material, e.g., an interface between two dissimilar waveguide arrays^15^. However, an interface can also separate two optical systems that differ only by the artificial gauge fields created in them. Generally, such a “gauge interface” marks a different dispersion curve on either side of the interface; hence, it must affect the transmission and reflection at the interface.

Gauge fields (GFs) are a basic concept in physics describing forces applied on charged particles. Artificial GFs are a technique for engineering the potential landscape such that neutral particles will mimic the dynamics of charged particles driven by external fields. With the advent of the particle-wave duality, artificial GFs have been demonstrated to act on photons^16–19^, cold atoms^20,21^, acoustic waves, etc. These artificial GFs are generated either by the geometry^17^ or by time-dependent modulation^18^ of system parameters. With the growing interest in topological systems^22^, which necessitate GFs^23,24^, it was suggested that the interface between two regions of the same medium but with different GFs in each region can create an effective edge. In these systems, both sides of the interface have the same basic dispersion properties, altered only by applying a different GF on each side. Such a gauge edge was employed to demonstrate analogies to the Rashba effect^25^, optical waveguiding^26,27^, topological edge states^28,29^ and back-refraction^30^. In the presence of a different GF on either side of the interface, the trajectories of waves crossing from one side to the other are governed by the symmetries in the system, which are expected to result in an effective Snell’s law, whereas the reflection and transmission coefficients arise from the specific boundary.

Here, we theoretically and experimentally demonstrate the effective Snell law governing the reflection and transmission of waves at an interface between regions of the same photonic medium, differing only in the artificial gauge fields introduced on either side. We show how the transverse momenta of the reflected and transmitted waves change according to the interfacial change in the gauge field, and demonstrate TIR and complete transmission. Subsequently, we provide an approximate calculation for the Fresnel coefficients for our example and explain how to generalize the concepts. Finally, we show how to concatenate several “gauge interfaces” and propose a design for a gauge-based imaging system—a device composed of several interfaces between different gauge fields, acting to reconstruct (image) an arbitrary paraxial input wavepacket at a predetermined plane.

## Results

**Fig. 1:**
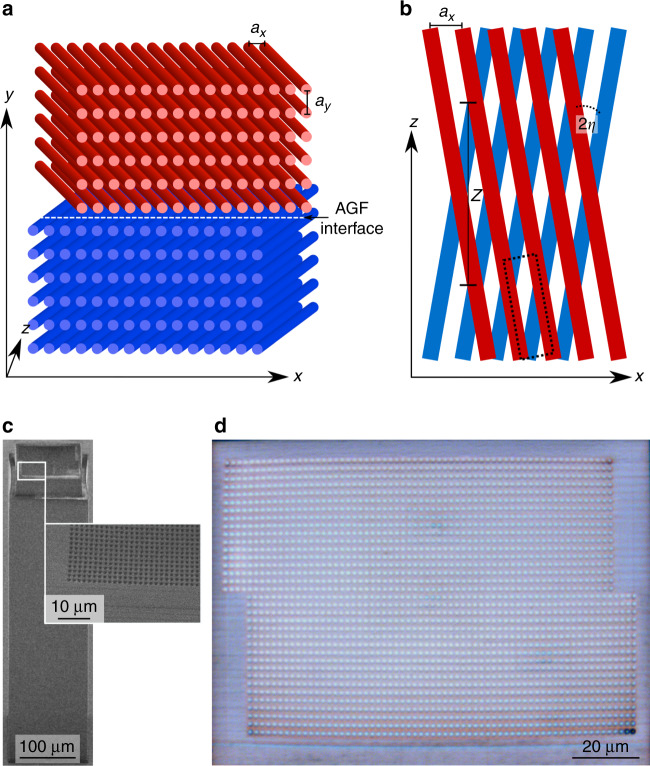
Sketch of our artificial gauge interface. Two rectangular arrays of waveguides (red: upper array, blue: lower array) are stacked on top of each other, creating an artificial GF interface in the *y*-direction. The waveguide arrays are tilted by 2*η* with respect to each other. Otherwise, the parameters for the two arrays are identical. **a** Front view. **b** Top view. The dashed box represents one unit cell in the *x–z-*plane in the upper array. **c** SEM image of the inverse fabricated waveguide sample from the side. The inset shows a magnified region to visualize the hollow waveguides. **d** Microscope image of the infiltrated sample from the top

For simplicity, consider first a simple system constituting an artificial gauge interface: two 2D arrays of evanescently coupled waveguides, where the waveguides in each array follow a different trajectory along the propagation axis *z* (Fig. 1). This model system serves to explain the ideas involved, which are later generalized. The GFs in our system are a direct result of the trajectories of the waveguides and do not require any temporal modulation of the materials at hand. The upper array experiences a constant tilt in the *x*-direction from the propagation axis *z*, with a paraxial angle *η* such that *x*(*z*) = *x*′ + *ηz* (paraxiality allows sin *η* ≃ *η*, where *x*′ is the original *x*-position), while the lower array is tilted by −*η* (Fig. 1a). These two arrays combined exhibit different artificial GFs that cannot be gauged away by a coordinate transformation. The propagation of light in this structure is described by the paraxial wave equation

1 $$i\partial _z\psi \left( {\vec r} \right) = - \frac{1}{{2k_0}}\nabla _ \bot ^2\psi \left( {\vec r} \right) - \frac{{k_0}}{{n_0}}{\Delta} n\left( {\vec r} \right)\psi \left( {\vec r} \right)$$

Here, *ψ* is the envelope of the electric field, *k*_0_ is the optical wavenumber inside the bulk material, *n*_0_ is the ambient refractive index, ${\Delta} n\left( {\vec r} \right) = n\left( {\vec r} \right) - n_0$ gives the relative refractive index profile, and $\nabla _ \bot ^2 = \partial _x^2 + \partial _y^2$. Eq. (1) is mathematically equivalent to the Schrödinger equation, where *z* plays the role of time, and ${\Delta} n\left( {\vec r} \right)$ plays the role of the potential. This analogy between the paraxial wave equation and the Schrödinger equation has been used many times in exploring a plethora of fundamental phenomena, ranging from Anderson localization^31^ and Zener tunneling^32^ to non-Hermitian potentials^33^ and Floquet topological insulators^23^.

The basic building block in our system is a two-dimensional array of evanescently coupled straight waveguides, i.e., ${\Delta} n\left( {\vec r} \right)$ is a periodic function in both *x* and *y*, with periods *a*_*x*_ and *a*_*y*_, respectively, such that each unit cell consists of a single waveguide.

Consider first an array where the trajectories of all the waveguides are in the *z*-direction. Following coupled mode theory^25^, the spectrum of light propagating in such a 2D array of waveguides is given by

2 $$\beta \left( {k_x,k_y} \right) = \beta _0 + 2c_x\cos \left( {k_xa_x} \right) + 2c_y\cos \left( {k_ya_y} \right)$$

where *β* is the propagation constant of an eigenmode, defined by $\psi \left( {x,y,z} \right) = \psi _0\left( {x,y} \right)e^{ - i\beta z}$, *k*_*x*_ and *k*_*y*_ are the spatial momenta of the mode in the *x*- and *y*-directions, *c*_*x*_ and *c*_*y*_ are the coupling strengths between adjacent waveguides in the *x*- and *y*-directions (taken to be real negative numbers according to standard solid state notation) and *β*_0_ is the propagation constant of the guided mode in a single isolated waveguide. Consider now an array of waveguides tilted at an angle *η* with respect to the *z*-axis such that the waveguides follow a trajectory defined by *x* − *ηz* = constant. The dynamics in an array of tilted waveguides are expressed by an artificial GF, given by the effective vector potential $\vec A\left( z \right) = - k_0\eta \hat x$, with the following spectrum^25,27^:

3 $$\beta _\eta \left( {k_x,k_y} \right) = \beta _0 + 2c_x\cos \left( {\left( {k_x - k_0\eta } \right)a_x} \right) + \eta k_x - \frac{1}{2}k_0\eta ^2 +\, 2c_y\cos \left( {k_ya_y} \right)$$

The shift of *k*_0_*η* in the cosine is the compensation due to the Galilean transformation of the waveguides. The linear *ηk*_*x*_ shift term appears because the spectrum in Eq. (3) is expressed in the laboratory frame and not in the frame of reference in which the waveguides are stationary. The constant offset $\frac{1}{2}k_0\eta ^2$ results from the effective elongation of the optical path inside the tilted waveguides.

Such a linear tilt of a waveguide array is, in itself, a trivial gauge field, as we can eliminate its effects by changing the frame of reference to the co-moving frame, i.e., a linear coordinate transformation of the entire system can gauge it out. This can also be understood by examining the arising effective magnetic field $\vec B = \vec \nabla \times \vec A$, which is zero for a constant vector potential $\vec A$. To have a nontrivial gauge, we need the effective gauge field to be nonuniform (i.e., have either space or time dependence). Such a nontrivial gauge is achieved by coupling two 2D arrays, each with a different tilt angle and therefore a different gauge. Then, it becomes impossible to gauge away the effect of the tilt when we combine two such fields with different tilts. There is no coordinate system in which both arrays would be simultaneously untilted^27^. Here ${\vec A}=\eta k_{0}{\widehat x}$ at the upper section, and ${\vec A}=-\eta k_{0}{\widehat x}$ at the lower section.

With this in mind, consider a two-dimensional array of evanescently coupled waveguides divided into two regions—top and bottom, as shown in Fig. 1a. The rows of waveguides in the top and bottom regions are identical in every parameter except for the tilt. The overall GF is then given by

4 $$\vec A\left( {\vec r} \right) = \left( {2{\Theta} \left( y \right) - 1} \right)\eta k_0\hat x$$

where Θ(*y*) is the Heaviside function, which is 1 when *y* > 0 and zero otherwise. This vector potential gives an effective magnetic field $\vec B = - 2\delta \left( y \right)\eta k_0\hat z$ with a ${\Phi} _B = - 2\eta k_0a_x$ magnetic flux through a unit cell at the interface.

The different gauge fields in each subsystem result in a different band structure (dispersion relation) for each subsystem. The system has discrete symmetries in both the *x*- and *z*-directions, with periods of *a*_*x*_ and $\frac{{a_x}}{{2\eta }}$, respectively (see Supplementary Information). Each of these dictates a conservation law for the respective momentum (up to 2*π* over the period), leaving only *k*_*y*_ to be modified as a wave crosses between the two regions. Thus, launching an eigenmode (a Bloch wave) with a defined wavevector (*k*_*x*_, *k*_*y*,inc_) on one side of the system will result in refraction and reflection of the wave upon incidence at the interface. According to the *x*- and *z*-translational symmetries of the joint lattice, the wavenumber in the second half plane will have to satisfy

5 $$\beta _\eta \left( {k_x,k_{y,{\mathrm{inc}}}} \right) = \beta _{ - \eta }\left( {k_x,k_{y,{\mathrm{tran}}}} \right)$$

where *k*_*y*,inc_ is the *y*-wavenumber of the incident beam, *k*_*y*,tran_ is the *y*-wavenumber of the transmitted beam, and *β*_*η*_(*k*_*x*_, *k*_*y*,inc_) is given by Eq. (3). Equation (5) acts as a generalized Snell law for an interface between two regions of the same medium but with different artificial gauge fields on each side in the specific realization of titled photonic lattices.

Note that Eq. (5) is general and valid for any interface that satisfies the symmetries in *x* and *z* (the plane normal to the interface), even for a uniform dielectric medium. The main difference between refraction from a dielectric interface and refraction from an AGF interface lies in the dispersion relation, that is, the relation between the propagation constant *β* and the frequency. In uniform dielectrics, a plane wave (which is an eigenmode of the medium) obeys $\beta _{{\mathrm{dielectric}}} = \sqrt {\left( {\frac{{n\omega }}{c}} \right)^2\, -\, (k_x^2 + k_y^2)}$. On the other hand, for an AGF medium, the dispersion can be designed as almost any desired relation^34,35^. In the specific case of tilted waveguide arrays, the dispersion relation is given by Eq. (3). The ability to design the dispersion allows for interesting dynamics such as a negative group velocity for some *k*_*x*_ values, for example, $2c_xa_x\sin \left( {\left( {k_x - k_0\eta } \right)a_x} \right) < -\! \eta$ for the case of tilted waveguide arrays. Engineering the dispersion relation also makes it possible to cancel diffraction in one of the directions, as we suggest in the “Discussion” section (Fig. 7). Notably, uniform dielectric interfaces require materials with different optical properties on each side of the interface, whereas an AGF interface can be engineered even when both sides have the same optical properties (up to their gauge), achieving refraction using the same materials of the same composition and configuration (periodicity).

**Fig. 2:**
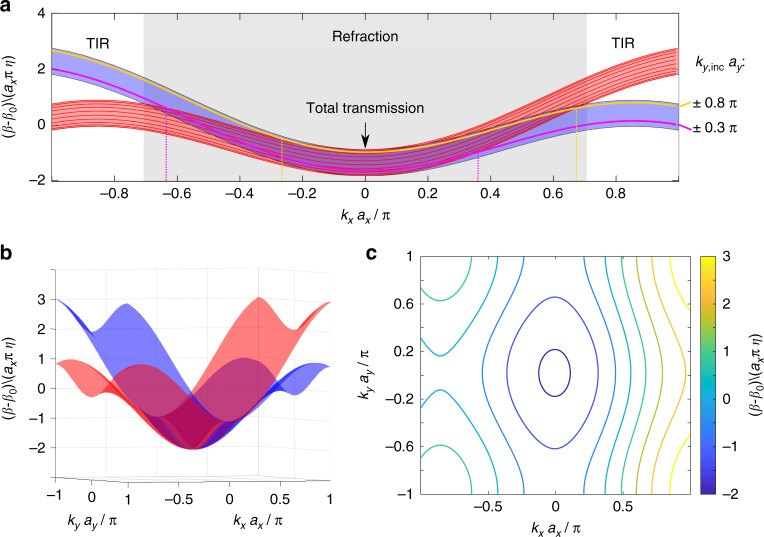
Dispersion relations for the upper (red) and lower (blue) arrays. **a** Projection of *β* as a function of *k*_*x*_. The solid lines mark some specific values of *k*_*y*_*a*_*y*_. In the *k*_*x*_-range where the two bands overlap, any wavepacket traveling across the artificial gauge interface undergoes refraction according to the generalized Snell law (shaded gray region). Outside this range, total internal reflection (TIR) occurs. Note that the *k*_*x*_-range for total reflection (unshaded region) is not necessarily symmetric around *k*_*x*_ = 0 (see vertical dashed lines, for a given *k*_*y*_). The parameters here are the same as in the experiment: *r* = 0.52 μm, *a*_*x*_ = 1.69 μm, *a*_*y*_ = 2.15 μm, *η* = 0.0093, and *λ* = 700 nm. **b** Full 3D dispersion relation *β* as a function of *k*_*x*_ and *k*_*y*_. **c** Contour plot of the equi-*β* over *k*_*x*_ and *k*_*y*_ for the red band. Each line represents an equi-*β* contour. The group velocity of a wavepacket at each point is perpendicular to the equi-*β* contour that goes through that point

Figure 2 shows the band structures for the upper (red) and lower (blue) waveguide arrays. Depicted in three dimensions (Fig. 2b), we note the sinusoidal shape of the dispersion along *k*_*x*_ as well as along *k*_*y*_. The projection onto the *k*_*x*_-component (Fig. 2a), however, helps us display the *k*_*y*_-conversion between the two arrays. In the projected band structure, each band represents the values of *β* (which plays the role of energy in the analogy to the Schrödinger equation) for all values of *k*_*y*_ associated with that band (see the Supplementary Information for a discussion on band replicas arising from the periodicity of the structure in *x* and *z*). As an example, the solid lines in Fig. 2a indicate the values of *β* associated with some specific *k*_*y*_. Note that the *k*_*x*_-range for total reflection (dashed vertical lines in Fig. 2a) is not necessarily symmetric around *k*_*x*_ = 0 (for a given *k*_*y*_). From this figure, it may seem that *β* is not periodic in *k*_*x*_, but a closer look at the symmetries in the system reveals that the periodicity is maintained (see the discussion in the Supplementary Information). Figure 2c displays a contour plot of equi-*β* as a function of *k*_*x*_ and *k*_*y*_. The group velocity of a wavepacket at each point is perpendicular to the equi-*β* contour that goes through that point.

When a wavepacket crosses the artificial GF interface between the lower array and the upper array, *β* is conserved such that $\beta _\eta \left( {k_x,k_{y,{\mathrm{inc}}}} \right) = \beta _{ - \eta }\left( {k_x,k_{y,{\mathrm{tran}}}} \right)$, as is the transverse wavenumber, *k*_*x*,inc_ = *k*_*x*,tran_ = *k*_*x*_. Graphically, this means that at this value of *β* the red and blue bands in Fig. 2a overlap. The quasi-energy *β* of the red band may belong to a different *k*_*y*_ than that of the blue band; thus, *k*_*y*,inc_ ≠ *k*_*y*,tran_ (see solid colored lines in Fig. 2a). Therefore, when the light crosses the artificial GF interface, *k*_y,inc_ must change according to

6 $$\cos (k_{y,{\mathrm{tran}}}\,a_y) - \cos \left( {k_{y,{\mathrm{inc}}}\,a_y} \right) = \frac{{c_x}}{{c_y}}\left[ {\cos \left( {\left( {k_x + k_0\eta } \right)a_x} \right) - \cos \left( {\left( {k_x - k_0\eta } \right)a_x} \right)} \right] - \eta \frac{{k_x}}{{c_y}}$$

**Fig. 3:**
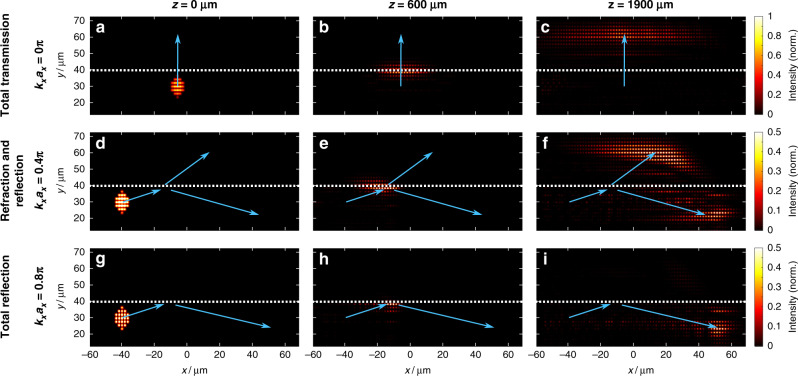
Simulated dynamics of beams in the three regimes. Total transmission (upper row), refraction and reflection (middle row), and total internal reflection (bottom row). Each panel shows the numerically calculated intensity distributions at three different *z*-values along the propagation direction: input facet (left column), *z* = 600 μm (middle column) and *z* = 1900 μm (right column). The dashed white line indicates the location of the gauge interface. The initial *y*-wavenumber is always *k*_*y*,inc_*a*_*y*_ = 0.5*π*. **a**–**c** An input beam with *k*_*x*_*a*_*x*_ = 0 is transmitted completely across the interface. **d**–**f** An input beam with *k*_*x*_*a*_*x*_ = 0.4*π* is partially reflected and partially refracted at the interface. The refraction is highlighted by the change in the trajectory. **g**–**i** An input beam with *k*_*x*_*a*_*x*_ = 0.8*π* undergoes total reflection, never crossing the interface. The intensity is normalized separately in each panel, and the intensity in the second and third rows is enhanced for better visibility. The arrows are guides to the eye and indicate the approximate trajectories of the respective beams

We identify three different regimes, which depend on *k*_*x*_:

1. Total internal reflection (TIR): *k*_*x*_ is such that the blue and red bands do not intersect, hence no coupling from the upper to the lower array (and vice versa) is possible. Consequently, the wavepacket is completely reflected (see Fig. 3g–i).
2. Perfect transmission for *k*_*x*_ = 0: A wavepacket crosses the interface between the two arrays without changing its wave vector components while allowing all the light to be transmitted through the interface (see Fig. 3a–c).
3. Refraction and reflection for all other values of *k*_*x*_: The red and blue bands intersect, but for different *k*_*y*,inc_ and *k*_*y*,tran_. As the “energy” *β* and the transverse wavenumber *k*_*x*_ are conserved, *k*_*y*_ has to change upon crossing the interface, resulting in both a refracted wave and a reflected wave (Fig. 3d–f).

Examples of the dynamics of waves in these three regimes are given in Fig. 3, which shows the results of direct simulations of Eq. (1) (using the commercial OptiBPM code), with parameters corresponding to those used in the experiments. The figure shows the intensity of optical beams at three different propagation planes along *z*. We probe the three different regimes by launching input beams with a set *k*_*y*,inc_ *a*_*y*_ = 0.5*π* and selecting *k*_*x*_*a*_*x*_ corresponding to total transmission (*k*_*x*_*a*_*x*_ = 0), refraction and reflection (*k*_*x*_*a*_*x*_ = 0.4*π*), and total internal reflection (*k*_*x*_*a*_*x*_ = 0.8*π*). Upon excitation at *z* = 0 μm (Fig. 3a, d, g), the beams travel toward the interface (indicated by the dashed white line), reaching it approximately after *z* = 600 μm (Fig. 3b, e, h). For the input beam with *k*_*x*_*a*_*x*_ = 0, the beam is completely transmitted across the interface (Fig. 3c) without any reflection. After passing through the interface, the beam strongly disperses (diffracts) in the *x*-direction. For the input beam with *k*_*x*_*a*_*x*_ = 0.4*π*, part of the beam is reflected by the interface, returning to the lower array, while part of it is refracted, as indicated by the change in the slope of the arrow (Fig. 3f). For the beam with *k*_*x*_*a*_*x*_ = 0.8*π*, the beam undergoes total reflection, never crossing the interface (Fig. 3i).

**Fig. 4:**
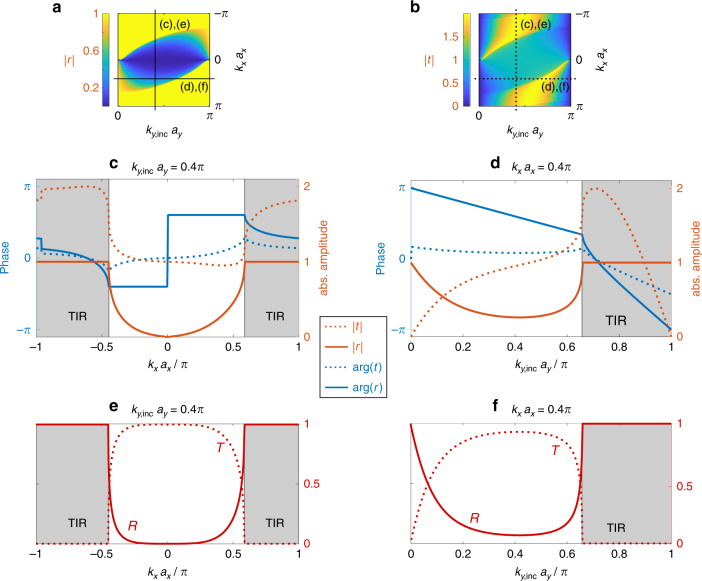
Fresnel coefficients. Reflection amplitude (**a**) and transmission amplitude (**b**) across the entire *k*_*x*_ and *k*_*y*_ range. The line cuts in the middle row show the phase (blue lines) and amplitude (red lines) of the reflection coefficient *r* (solid lines) and the transmission coefficient *t* (dashed lines) along the cuts indicated in (**a**) and (**b**): for *k*_*y*,inc_*a*_*y*_ = 0.4*π* (**c**) and *k*_*x*_*a*_*x*_ = 0.4*π* (**d**). Note that |*t*| can be greater than 1 for some (*k*_*x*_, *k*_*y*,inc_). **e** and **f** show the reflectance *R* = |*r*|^2^ (solid line) and transmittance $T = Re\left\{ {\frac{{v_{gy,{\mathrm{tran}}}}}{{v_{gy,{\mathrm{inc}}}}}} \right\}\left| t \right|^2$ (dashed line) for the same wave vectors as in (**c**) and (**d**). The total energy flux is conserved, as *R* + *T* = 1 holds for all regions

Having used symmetry and the dispersion relation to find the general laws of refraction and reflection at a gauge interface, the next step is natural: finding the coefficients for reflection and transmission. However, similar to the Fresnel coefficients at a dielectric interface, this calculation is system specific, and the details depend on the interface between the two regions. That is, unlike the Snell-like law, the Fresnel coefficients cannot be generalized (conservation of power yields a relation between the absolute values of the Fresnel coefficients, but to obtain the coefficients, one must also employ continuity at the interface). With this in mind, we calculate the Fresnel-like coefficients for our example of a gauge interface constructed from titled waveguides. We use an approximate model^27^ for the coupling between the two sections and derive an approximate formula for the coefficients. The details of the calculation are presented in the Supplementary Information. Figure 4 shows the Fresnel-like coefficients for our gauge interface for parameters corresponding to those shown in Fig. 3. We plot the amplitude and phase of the transmitted and reflected parts for *k*_*y*,inc_ *a*_*y*_ = 0.4*π* as a function of *k*_*x*_ and for *k*_*x*_*a*_*x*_ = 0.4*π* as a function of *k*_*y*,inc_ (Fig. 4c, d, respectively). As explained above, the Fresnel coefficients are highly dependent on the specifics of the interface; hence, a different model for the gauge field interface will yield different coefficients. However, the Snell-like law of refraction will not change, as it depends only on the symmetry and the dispersion relations on both sides of the interface.

**Fig. 5:**
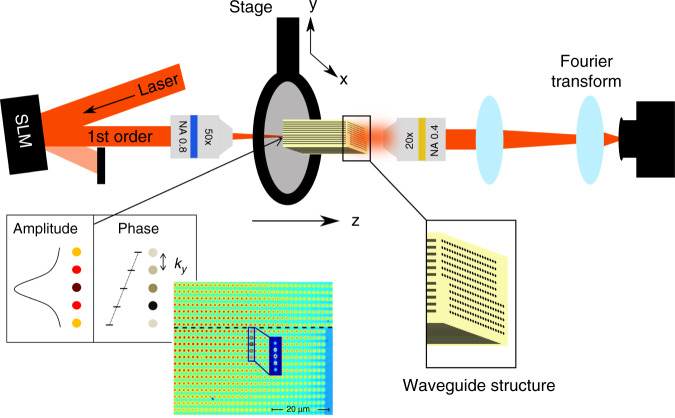
Experimental setup. A laser beam (wavelength 700nm) is reflected off an SLM, which imprints a specific phase and amplitude pattern onto the beam. To shape the amplitude while using a phase-only SLM, we overlay the phase pattern on the SLM with a blazed grating that shifts parts of the reflected light into the first diffraction order^37^. After Fourier transform by an objective, the beam consists of five spots with a phase difference of *k*_*y*,inc_*a*_*y*_ between each of them and a Gaussian amplitude envelope (inset). These spots are focused onto a row of waveguides below the interface, as shown by the false color photograph of the beam on top of the sample, with the interface marked by the dashed line. The light then propagates along *z*, interacts with the interface, and exits the sample after a propagation distance of *z* = 725 μm. The intensity distribution at the output facet is imaged by a camera, as is its spatial power spectrum (obtained by inserting an extra lens at the focal distance to the camera)

To demonstrate the generalized Snell’s law in experiments, we fabricate sets of tilted optical waveguides corresponding to the system described in Fig. 1. The samples are fabricated using direct laser writing to create hollow waveguides, which are subsequently infiltrated with a higher index material (Fig. 1c, d). For details on the fabrication, see ref. ^36^. The experimental measurement setup is sketched in Fig. 5. We reflect a 700 nm laser beam off a spatial light modulator (SLM) to excite a Bloch mode with a given *k*_*y*,inc_ while exciting the entire first Brillouin zone in *x* (i.e., −*π* ≤ *k*_*x*_*a*_*x*_ ≤ *π*). To do this, the beam reflected off the SLM^37^ is shaped such that after a Fourier transform (by a microscope objective), it consists of 5 lobes, with their phase forming a linear ladder, commensurate with the chosen Bloch mode, while in *x*, the beam is simply focused into a single row of waveguides. The beam passes through the sample, and the output facet is imaged by a camera. Since we are interested in investigating the passage through the gauge interface, we scan the value of *k*_*y*,inc_ by changing the relative phase between the lobes while exciting the entire first Brillouin zone in *x*. Figure 5 shows a false color photograph of the input beam overlay a photograph of the sample, with the interface marked by the dashed line. The light propagates across the interface, and we measure the intensity of the refracted and reflected waves at the output facet of the sample, as well as the intensity at the Fourier plane (obtained at the focal plane of a lens), which corresponds to the spatial power spectrum.

**Fig. 6:**
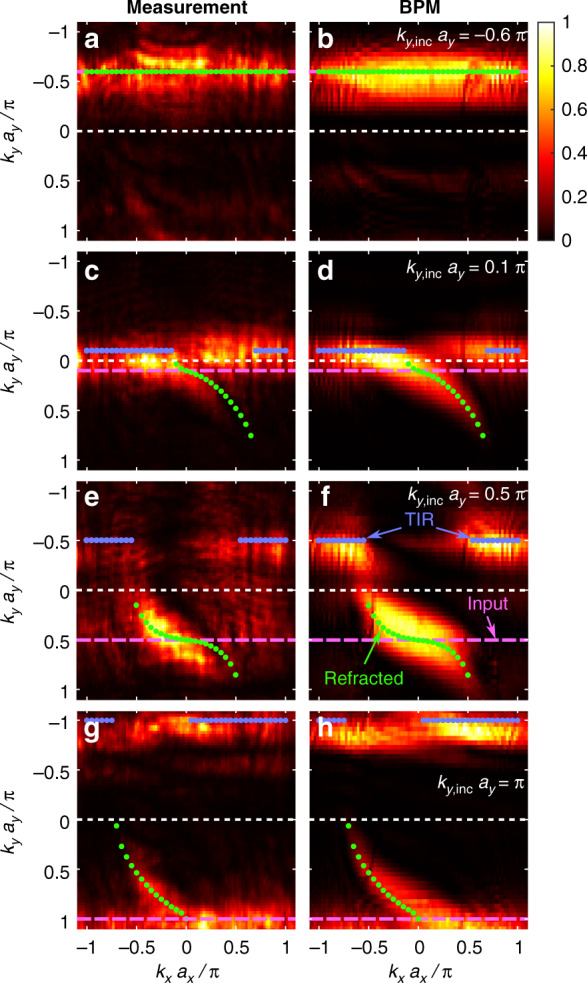
Refraction and reflection by an artificial gauge interface. Spatial power spectrum (intensity in Fourier space) for an input wave with several values of *k*_*y*,inc_ always launched at the same position in *y*. The left and right columns depict the experimental and simulated results, respectively. The purple dashed line shows the location of the input beam in Fourier space and the dots show the values analytically calculated from Eq. (6) (green for refraction and blue for TIR). For *k*_*y*,inc_*a*_*y*_ = −0.6*π*, the beam travels away from the interface and does not refract at all (**a**, **b**), while for the other panels (**c**–**h**), we see both partial refraction and reflection. In the second row (**c**, **d**), the beam is launched with *k*_*y*_*a*_*y*_ = 0.1*π*. As these panels show, the output beam is split: for −0.1*π* < *k*_*x*_*a*_*x*_ < 0.6*π*, the beam is transmitted and displays distinct refraction, according to the generalized Snell’s law expressed by Eq. (6), while in the regions beyond this range, the beam experiences TIR. Note the prominent asymmetry between the minimal and maximal *k*_*x*_ boundaries between the regions of transmission and TIR. In the third row (**e**, **f**), the beam is launched with *k*_*y*_*a*_*y*_ = 0.5*π*. For −0.5*π* < *k*_*x*_*a*_*x*_ < 0.5*π*, the beam is transmitted, while beyond this range, the beam experiences TIR. The experiment (**e**) captures both the refraction and TIR regions. In the 4th row, (**g**, **h**), the beam is launched with *k*_*y*_*a*_*y*_ = *π*. For −0.7*π* < *k*_*x*_*a*_*x*_ < 0.1*π*, the beam is transmitted. Beyond this domain, the beam experiences TIR. Here, both the incident and reflected beams have the same |*k*_*y*_|. Altogether, the comparison of the measurements (left row), simulations (right row), and analytical expression from Eq. (6) (green and blue dots) shows good agreement with the expected k_*y*,tran_-distribution. A movie showing the complete set of measurements can be found in Supplementary Movie no. #1.

Figure 6 shows the spatial power spectrum (Fourier space intensity) of the waves exiting the sample for an input wave with different *k*_*y*,inc_ values but always launched at the same position in *y*, along with a comparison to beam-propagation simulations. The analytically calculated values for *k*_*y*,tran_ obtained by Eq. (6) are marked by the green and blue dots on top of the experimental and simulated results. The beam travels toward the artificial GF interface with a group velocity *v*_*gy*_, obtained from the dispersion relation in Eq. (3) by taking the derivative with respect to *k*_*y*_*a*_*y*_. In the first row (Fig. 6a, b), the beam is launched such that it moves away from the interface (*k*_*y*_*a*_*y*_ = −0.6*π*), and never reaches the interface; hence, the output beam has the same spatial spectrum as the input beam. As Fig. 6a, b show, the power spectrum of the output beam is located around the same *k*_*y*_*a*_*y*_ = −0.6*π* as the input beam. In the second row (Fig. 6c, d), the beam is launched with *k*_*y*_*a*_*y*_ = 0.1*π*. As these panels show, the output beam is split: for −0.1*π* < *k*_*x*_*a*_*x*_ < 0.6*π*, the beam is transmitted and displays distinct refraction, according to the generalized Snell’s law expressed by Eq. (6), while in the regions beyond this range, the beam experiences TIR. Note the prominent asymmetry between the minimal and maximal *k*_*x*_ boundaries between the regions of transmission and TIR. In experiment (c), the measurement only partially shows the results, as the group velocity in *y* for *k*_*y*_*a*_*y*_ = 0.1*π* is very low, and the beam has only partially passed the interface even upon reaching the output facet of the sample. In the third row, Fig. 6e, f, the beam is launched with *k*_*y*_*a*_*y*_ = 0.5*π*. For −0.5*π* < *k*_*x*_*a*_*x*_ < 0.5*π*, the beam is transmitted, while beyond this range, the beam experiences TIR. Here, the asymmetry between the minimal and maximal *k*_*x*_ boundaries between transmission and TIR is less significant. The experiment (Fig. 6e) captures both the refraction and TIR regions. In the fourth row, Fig. 6g, h, the beam is launched with *k*_*y*_*a*_*y*_ = *π*. For −0.7*π* < *k*_*x*_*a*_*x*_ < −0.1*π*, the beam is transmitted. Beyond this domain, the beam experiences TIR. Here, both the incident and reflected beams have the same |*k*_*y*_|. This case is similar to prism coupling at a grazing angle to couple a beam into a waveguide where it will be bound by TIR.

The comparison of the experimental measurements and numerical calculations with the analytical Eq. (6) (dots in Fig. 6) shows good agreement for the refracted part. As expected, the obtained *k*-distribution is broader than the analytical curve due to finite size effects. Namely, the input beam with *k*_*y*,inc_ has a finite width (see Fig. 5) of five waveguides. The more waveguides are excited in real space, the smaller the width of the *k*-component in Fourier space. However, we do not want the input pattern to excite waveguides in the other array across the artificial GF interface; hence, we have to limit the size of the input beam. In addition, the center position of the input pattern needs to be close enough to the artificial GF interface such that the beam can travel across the artificial GF interface in the given propagation distance. Therefore, we need to limit the number of excited waveguides. For excitation of five waveguides, the width is Δ*k*_*y*,inc_ *a*_*y*_ ≈ 0.4*π* (see Fig. 6). As the same number of illumination spots is chosen in the experiments, the numerical calculation reflects the experimental conditions very well. Altogether, the comparison of the measurements, simulations, and analytical expression shows good agreement with the expected *k*_*y*,tran_-distribution. A movie showing the complete set of measurements can be found in Supplementary Movie #1.

## Discussion

**Fig. 7:**
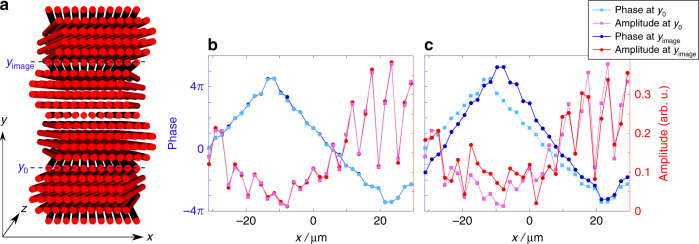
Gauge-based imaging system. **a** Sketch of a gauge-based imaging system. The tilt angle *η* for each row as a function of *y* is given by $\eta \left( y \right) = \eta _0\sin \left( {\frac{{2\pi \left( {y - y_0} \right)}}{{y_{{\mathrm{image}}} - y_0}}} \right)$. We launch the beam at *y*_0_ and expect it to reconstruct at *y*_image_. In the calculations, we assume a periodicity in *x* of 32 waveguides. **b**, **c** The amplitude (pink) and phase (light blue) at *y* = *y*_0_ and *z* = 0 (squares) compared to the amplitude (red) and phase (dark blue) at *y* = *y*_image_ (circles). **b** The system parameters satisfy Eq. (8), and reconstruction in both the amplitude and phase is achieved. The output distance *z* is chosen such that the maximal total power is obtained at the image plane (which occurs at *z* = 1000 μm). **c** The system parameters are the same as in (**b**) except for *η*_0_ such that now, it does not satisfy Eq. (8). Even at the *z* with the best fit by cross-correlation (*z* = 926 μm), the original signal differs completely from the output signal

Having demonstrated the Snell law for refraction and reflection at an interface between two different artificial GFs, we move on to concatenating several gauge interfaces and constructing devices. As an example highlighting the possibilities that refraction by artificial GFs allows, we design a gauge-based imaging system. By realizing such a system with arrays of tilted waveguides, we design a scheme that maps any (arbitrary) wavepacket input at the input facet to the output facet. In the scheme based on waveguide arrays, this corresponds to a system with different rows of waveguides tilted at different angles (Fig. 7a) mapping an input state from row *y*_0_ to row *y*_image_. For every row of waveguides with a tilt *η*(*y*) positioned at *y*, we find the propagation constant $\beta _{\eta \left( y \right)} = \beta _0 + 2c_x\cos \left( {\left( {k_x - k_0\eta \left( y \right)} \right)a_x} \right) + \eta \left( y \right)k_x - \frac{1}{2}k_0\eta ^2\left( y \right).$ To produce an image, we need the phase accumulation by all components to be identical. Thus, we require that when a wavepacket diffracts along *y* and propagate along *z* from input row *y*_0_ to output row *y*_image_, the cumulative phase accumulation for each of its *k*_*x*_ constituents is the same. Therefore, the value of ${\int}_0^{y_{{\mathrm{image}}}} {\beta _{\eta \left( y \right)}} \left( {k_x} \right){\mathrm{d}}y$ should not depend on *k*_*x*_. This can be written as

7 $$\frac{{\mathrm{d}}}{{{\mathrm{d}}\left( {k_x} \right)}}{\int_0^{y_{{\mathrm{image}}}}} {\beta _{\eta \left( y \right)}} \left( {k_x} \right){\mathrm{d}}y = 0$$

This requirement can be fulfilled in a 2D array of straight waveguides, where each row (*y*) is tilted at a different angle such that$\eta \left( y \right) = \eta _0\sin \left( {\frac{{2\pi y}}{{y_{{\mathrm{image}}}}}} \right)$. The requirement in Eq. (7) can be expressed by

8 $${\int\nolimits_0^{2\pi }} {\exp } \left( { - ik_0\eta _0a_x\sin \left( {y^\prime } \right)} \right){\mathrm{d}}y^\prime = J_0\left( {k_0\eta _0a_x} \right) = 0$$

where *J*_0_ is the zeroth-order Bessel function and $y^\prime = \frac{{2\pi y}}{{y_{{\mathrm{image}}}}}$ is now unitless. In other words, by designing the tilt of each row properly, an arbitrary wavepacket *f*(*x*) at row *y* = 0 is reproduced at row *y*_image_ (apart from a global phase). Figure 7a shows a sketch of the gauge-imaging waveguide structure. Each row has a different tilt angle $\eta \left( y \right) = \eta _0\sin \left( {\frac{{2\pi \left( {y - y_0} \right)}}{{y_{{\mathrm{image}}} - y_0}}} \right)$. Figure 7b compares the amplitude and phase of the wavepacket launched at *y* = *y*_0_ and *z* = 0 (pink and light blue) to those of the imaged one at *y* = *y*_image_ and *z* = 1000 μm (red and dark blue), revealing that the final and initial wavepackets are essentially the same. One should note that the beam diffracts in *y* as it propagates along *z*; hence, the imaging is one-dimensional for the field distribution in *x* only. Therefore, the intensity reaching the row at *y*_image_ is limited by the 1D discrete diffraction in *z* such that the intensity at each row (neglecting back reflections) is given approximately by $| {J_{\frac{{{\mathrm{{\Delta} }}y}}{{a_y}}}\left( {f\left( z \right)} \right)}|^2$, where *f*(*z*) is a function of *z* that depends on the details of the coupling and $J_{\frac{{{\mathrm{{\Delta} }}y}}{{a_y}}}$ is the Bessel function of the order of row number $\frac{{{\mathrm{{\Delta} }}y}}{{a_y}}$. In our simulated example, there are 29 rows of waveguides between *y*_0_ and *y*_image_, so the maximal intensity that can propagate to *y*_image_ is limited to $\max \left| {J_{29}} \right|^2 \approx 4.7\%$ of the initial intensity. In practice, we obtain approximately 2.9% due to back reflections (in *y*) and slightly different effective couplings along *y* for each *k*_*x*_ component. In the simulation, we assume that the structure is periodic in *x* with a period of 32 waveguides. The output facet is chosen to support maximal total power at the imaging row. Figure 7c uses the same system as Fig. 7b, changing only the size of *η*_0_ such that Eq. (8) is no longer fulfilled. We simulate the propagation of the same wavepacket in this (non-imaging) structure up to the distance that gives the maximal cross-correlation between the input and the output, which occurs at *z* = 926 μm, yet it is clear that the input and output wavepackets do not overlap. Essentially, with proper design of this simple gauge-based imaging system, we can transfer an arbitrary wavepacket from an initial row at the input facet of the structure to a preselected row at the output facet. This idea works well as long as the Bloch mode spectrum in *x* never projects onto evanescent modes (generated by TIR) throughout the propagation. This example, albeit simple, demonstrates that it is possible to construct various optical devices and systems by engineering artificial gauge fields using just one dielectric material.

To summarize, we derived the laws of refraction and reflection at an interface between two regions differing solely by their artificial gauge fields and demonstrated the concepts in experiments in 3D-micro-printed optical waveguide arrays. Generalizing the concepts of refraction and reflection at a gauge interface offers exciting possibilities for routing light and more generally for constructing photonic systems in a given medium strictly by designing the local gauge. As an example, we proposed an imaging system that maps any input state from one place at the input facet to a predesignated other location at the output facet by cascading different artificial gauge fields.

## Supplementary information

SUPPLEMENTARY INFORMATION for Generalized Laws of Refraction and Reflection at Interfaces between Different Photonic Artificial Gauge Fields

Supplemental Video 1

## Data Availability

All experimental data and any related experimental background information not mentioned in the text are available from the authors upon reasonable request.
