# Supplementary material for: Generalized laws of refraction and reflection at interfaces between different photonic artificial gauge fields
Source: Light Sci Appl. 2020 Dec 22;9:200. doi: 10.1038/s41377-020-00411-7 (PMC7755922; doi:10.1038/s41377-020-00411-7)
Supplement: Supplementary file 1 — SUPPLEMENTARY INFORMATION for Generalized Laws of Refraction and Reflection at Interfaces between Different Photonic Artificial Gauge Fields [file 41377_2020_411_MOESM1_ESM.docx]

**SUPPLEMENTARY INFORMATION for**

**Generalized Laws of Refraction and Reflection at
Interfaces between Different Photonic Artificial Gauge Fields**

Moshe-Ishay Cohen^*1,2^, Christina Jörg^*3,4^, Yaakov Lumer^1,2^, Yonatan Plotnik^1,2^, Erik H. Waller^3^, Julian Schulz^3^, Georg von Freymann^3,5^ and Mordechai Segev^1,2^

^1^Physics Department, Technion – Israel Institute of Technology, Haifa 32000, Israel
^2^Solid State Institute, Technion – Israel Institute of Technology, Haifa 32000, Israel
^3^Physics Department and Research Center OPTIMAS, TU Kaiserslautern, 67663 Kaiserslautern, Germany

^4^Department of Physics, The Pennsylvania State University, Pennsylvania 16802, USA
^5^Fraunhofer Institute for Industrial Mathematics ITWM, 67663 Kaiserslautern, Germany

* Equal contribution

[msegev@technion.ac.il](mailto:msegev@technion.ac.il)

1. Sample fabrication:

The sample is fabricated in several steps. First, the structure surrounding the waveguides (i.e., the “inverse” of the waveguide sample) is fabricated using direct laser writing, a 3D-micro printing technique^1^. After subsequent development in propylene glycol methyl ether acetate and isopropanol, the sample comprises empty channels, surrounded by polymerized IP-Dip (Nanoscribe) with a refractive index of 1.54^2^. This sample is then infiltrated with the photo-resist SU8, to create the actual waveguides (refractive index 1.59)^3,4^. To solidify the SU8, the sample is baked on a hotplate for 3 minutes at 150°C. Figures 1(c) and (d) in the main text show a scanning electron microscope (SEM) image of the inverse sample and a microscope image of the infiltrated sample, respectively.

The parameters of the sample are:

Radius of waveguides $r=(0.52\pm0.07) \mu m$, distance between waveguides $a_{x}=(1.69\pm0.02) \mu m$, $a_{y}=(2.15\pm0.02) \mu m$, tilt $\eta=0.0093\pm0.0004$, and maximum propagation length corresponding to the height of the sample $h=\left( 725\pm3 \right) \mu m.$

1. Measurements:

The experimental set up was discussed in Fig. 4 in the main text. In this section, we explain how we carry out the measurements and handle the noise in the measurements.

The Fourier images in the measurements look 'stripy'. This is due to small inaccuracies in the position of the waveguides due to the fabrication. These inaccuracies cause noise in form of stripes in our data. Even small errors in the positions of the waveguides (< 25 nm) causes noise in the Fourier image, as the Fourier intensity (the spatial power spectrum) will reside at slightly shifted $k$-values. We estimate the maximum error in the positions of the waveguides from SEM measurements to be about 20 nm in the *x*- and *y*-direction. To visualize the influence of these errors on the Fourier-space intensity measurements, we simulate the propagation in the system with the OptiBPM code, where we shift the real-space positions of the waveguides in both *x*- and *y*-directions, with shifts drawn from a random normal distribution number in a given interval. Figure S1 shows the simulated influence of these position errors on the Fourier images. Specifically, (a) shows the Fourier intensity with all waveguides accurately positioned, whereas (b) and (c) show the Fourier intensity in the presence of two-dimensional random shifts in the intervals $\left[ -20 \mathrm{nm}, 20 \mathrm{nm} \right]$ and $\left[ -50 \mathrm{nm}, 50 \mathrm{nm} \right]$, respectively. Panel (d) shows how we can eliminate the influence of these random shifts: we average over an ensemble of 10 images for each value of $k_{y,\mathrm{inc}}$ at different positions along *x*. Panel (d) displays the result of the median, which clearly almost fully coincides with panel (a) – which does not include the random shifts. Hence, Fig. 5 in the main text displays the experimental results processed in this manner.

In addition to the fabrication-induced deficiencies, any tiny tilt due to misalignment of the input beam results in a shift of $k_{x}$ or $k_{y,\mathrm{inc}}$with respect to the input value we aimed at, which is determined by the pattern superimposed on the beam by the SLM. To counteract such misalignment-based errors, in addition to the Fourier images, we also take the real-space images during the calibration procedure. From them, we determine the movement of the center of the wavepacket. As this depends on the group-velocity $v_{g x,y}=\frac{\partial\beta}{\partial k_{x,y}}$ it shows a sinusoidal dependence on the $k_{x}$ ($k_{y}$) value. Therefore, we fit the measured value of the wavepacket's center of mass, ${COM}_{x,y}$, along $x (y)$ with ${COM}_{x,y}=\sin(k_{x,y}a_{x,y}+\phi_{x,y})$ to obtain the shift $\phi_{x,y}$ in the respective $k$-component. We then take this shift into account when plotting the Fourier images along the calibrated axes.


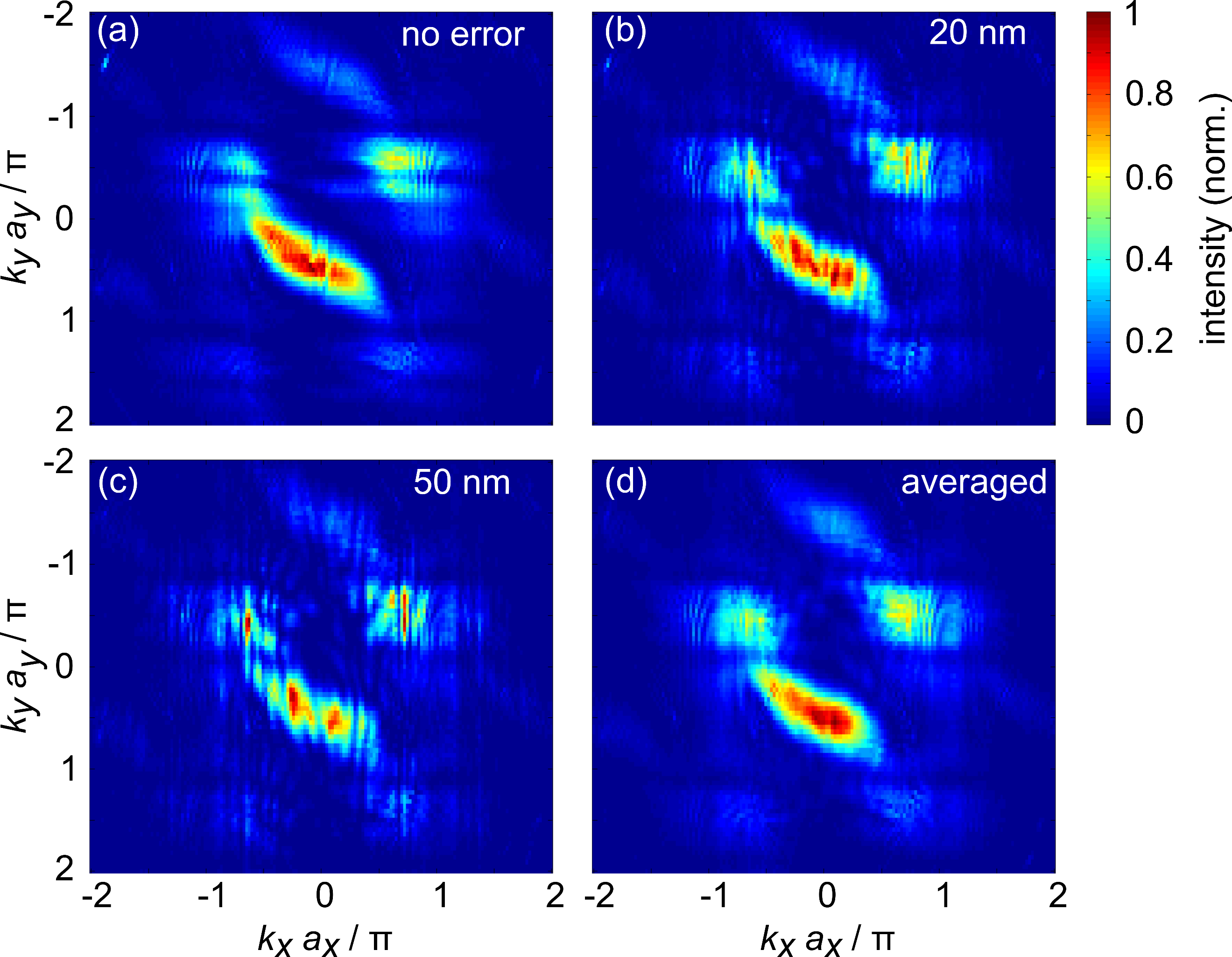


**Figure S1: Influence of position error on the Fourier image,** OptiBPM calculations. (a) Fourier image without error. (b) Fourier images in the presence of two-dimensional position errors drawn randomly in the intervals [-20 nm, 20 nm] and [-50 nm, 50 nm] (c), respectively. (d) Fourier image obtained by averaging over 6 Fourier images with position error of maximum 50 nm.

1. Fresnel coefficients for reflection and transmission:

The Fresnel coefficients, for our scheme of realizing an artificial gauge interface via tilted waveguides are calculated by considering an incident and reflected plane wave at the lower side of the interface ($y<0$) and a transmitted plane wave at the upper side of the interface ($y>0$). Keeping in mind that $\beta$ and $k_{x}$ are conserved^5^ (as explained in the main text), this yields

|  | $\psi\left( y;k_{x},k_{y,\mathrm{inc}} \right)=\left\{ \begin{matrix} te^{\left. -ik_{y,\mathrm{tran}}y \right.} & y>0 \\ re^{\left. ik_{y,inc}y \right.}+e^{\left. -ik_{y,inc}y \right.} & y\leq0 \end{matrix} \right\}e^{-i\beta_{\eta}\left( k_{x},k_{y} \right)z-ik_{x}x}$ | **(S1)** |
| --- | --- | --- |

where $r$ and $t$ are the complex reflection and transmission coefficients, respectively. At the interface, the coupling coefficient in the $y$-direction is changed, as the waveguides are tilted against each other. Therefore, the Hamiltonian at the interface looks like (see also the supplementary of Ref.^6^):

$$i\partial_{z}\left( \begin{matrix} \psi_{>0} \\ \psi_{<0} \end{matrix} \right)=i\partial_{z}\left( \begin{matrix} \vdots\\ \begin{matrix} te^{\left. -ik_{y,\mathrm{tran}}(2a_{y}) \right.} \\ te^{\left. -ik_{y,\mathrm{tran}}\left( a_{y} \right) \right.} \\ r+1 \\ re^{\left. ik_{y,inc}\left( -a_{y} \right) \right.}+e^{\left. -ik_{y,inc}\left( -a_{y} \right) \right.} \end{matrix} \\ \vdots\end{matrix} \right)e^{-i\beta_{\eta}\left( k_{x},k_{y,\mathrm{inc}} \right)z}=\left( \begin{matrix} \ddots& \vdots& 0 \\ \cdots& \begin{matrix} c_{y} & \beta_{-\eta}\left( k_{x} \right) & c_{+\to-} & 0 \\ 0 & c_{-\to+} & \beta_{+\eta}\left( k_{x} \right) & c_{y} \end{matrix} & \cdots\\ 0 & \vdots& \ddots\end{matrix} \right)\left( \begin{matrix} \vdots\\ \begin{matrix} te^{\left. -ik_{y,\mathrm{tran}}(2a_{y}) \right.} \\ te^{\left. -ik_{y,\mathrm{tran}}\left( a_{y} \right) \right.} \\ r+1 \\ re^{\left. ik_{y,inc}\left( -a_{y} \right) \right.}+e^{\left. -ik_{y,inc}\left( -a_{y} \right) \right.} \end{matrix} \\ \vdots\end{matrix} \right)e^{-i\beta_{\eta}\left( k_{x},k_{y,\mathrm{inc}} \right)z}$$

**(S2)**

The grey-shaded terms mark the sites at both sides of the interface. Here, $c_{y}$ is the coupling in the $y$-direction between two adjacent rows which move together with the same tilting angle, which depends weakly on $k_{x}$ (not written explicitly). $c_{+\to-}\approx C_{\mathrm{eff}}e^{-\frac{\sigma^{2}}{4}\left( k_{x}-A \right)^{2}}$ is the coupling at the interface from the array tilted with $\eta$ to the array tilted with$-\eta$, with the gauge field being $A=-k_{0}\eta$, given that $C_{\mathrm{eff}}$ and $\sigma$ are parameters which are numerically approximated following the supplementary of Ref.^6^. In the same vein, $c_{-\to+}\approx C_{\mathrm{eff}}e^{-\frac{\sigma^{2}}{4}\left( k_{x}+A \right)^{2}}$ is the coupling from the array tilted with $-\eta$ to the array tilted with $\eta$, $\beta_{\eta}\left( k_{x} \right)=\beta_{0}+2c_{x}\cos\left( \left( k_{x}-k_{0}\eta\right)a_{x} \right)+\eta k_{x}-\frac{1}{2}k_{0}\eta^{2}$ is the propagation constant of a single row tilted by $\eta$ with momentum $k_{x}$, $\beta_{\eta}\left( k_{x},k_{y} \right)=\beta_{\eta}\left( k_{x} \right)+2c_{y}\cos\left( k_{y}a_{y} \right)=\beta_{0}+2c_{x}\cos\left( \left( k_{x}-k_{0}\eta\right)a_{x} \right)+\eta k_{x}-\frac{1}{2}k_{0}\eta^{2}+2c_{y}\cos\left( k_{y}a_{y} \right)$ is the propagation constant of a 2D array tilted with $\eta$ in the $x$- direction and having momentum $\vec{k}=\left( k_{x},k_{y} \right)$.

Solving for the two rows at the interface for $r$ and $t$ yields:

$\left( \begin{matrix} te^{\left. -ik_{y,\mathrm{tran}}\left( a_{y} \right) \right.} \\ r+1 \end{matrix} \right)\beta_{\eta}\left( k_{x},k_{y,\mathrm{inc}} \right)=\left( \begin{matrix} te^{\left. -ik_{y,\mathrm{tran}}\left( a_{y} \right) \right.}\beta_{-\eta}\left( k_{x},k_{y,\mathrm{tran}} \right) \\ (r+1)\beta_{+\eta}\left( k_{x},k_{y,\mathrm{inc}} \right) \end{matrix} \right)=\left( \begin{matrix} te^{\left. -ik_{y,\mathrm{tran}}\left( a_{y} \right) \right.}\left[ \beta_{-\eta}\left( k_{x} \right)+2c_{y}\cos\left( k_{y,\mathrm{tran}} \right) \right] \\ \left( r+1 \right)\left[ \beta_{+\eta}\left( k_{x} \right)+2c_{y}\cos\left( k_{y,\mathrm{inc}} \right) \right] \end{matrix} \right)=\left( \begin{matrix} c_{y}te^{\left. -ik_{y,\mathrm{tran}}(2a_{y}) \right.}+\beta_{-\eta}\left( k_{x} \right)te^{\left. -ik_{y,\mathrm{tran}}\left( a_{y} \right) \right.}+c_{+\to-}\left( r+1 \right) \\ c_{-\to+}te^{\left. -ik_{y,\mathrm{tran}}\left( a_{y} \right) \right.}+\beta_{+\eta}\left( k_{x} \right)\left( r+1 \right)+c_{y}\left( re^{\left. ik_{y,inc}\left( -a_{y} \right) \right.}+e^{\left. -ik_{y,inc}\left( -a_{y} \right) \right.} \right) \end{matrix} \right)$ **(S3)**

After some algebra this results in:

|  | $r=\frac{\left. \frac{c_{-\to+}}{c_{y}}e^{\left. -ik_{y,\mathrm{tran}}\left( a_{y} \right) \right.}-\frac{c_{y}}{c_{+\to-}}e^{-\left. ik_{y,inc}\left( a_{y} \right) \right.} \right.}{\left. -\frac{c_{-\to+}}{c_{y}}e^{\left. -ik_{y,\mathrm{tran}}\left( a_{y} \right) \right.}+\frac{c_{y}}{c_{+\to-}}e^{\left. +ik_{y,inc}\left( a_{y} \right) \right.} \right.}$ | **(S4)** |
| --- | --- | --- |

|  | $t=\frac{c_{+\to-}}{c_{y}}\left( r+1 \right)=\frac{2i\sin\left( k_{y,\mathrm{inc}}a_{y} \right)}{\left. -\frac{c_{-\to+}}{c_{y}}e^{\left. -ik_{y,\mathrm{tran}}\left( a_{y} \right) \right.}+\frac{c_{y}}{c_{+\to-}}e^{\left. +ik_{y,inc}\left( a_{y} \right) \right.} \right.}$ | **(S5)** |
| --- | --- | --- |

To obtain energy conservation, we need to calculate the absolute reflection *R* and transmission *T*. Following Ref.^5^ we obtain

| $R=\left\vert r \right\vert^{2}.$ | **(S6)** |
| --- | --- |

For *T*, we need to consider the differences in group velocities between the upper and the lower parts (similar to conservation of energy flux at a simple dielectric interface). We get

| $T=\frac{v_{g,\mathrm{trans}}}{v_{g,\mathrm{inc}}}\left\vert t \right\vert^{2}=\frac{{\partial_{k_{y}}\beta}_{\eta}\left( k_{x},k_{y} \right)_{k_{y}=k_{\mathrm{trans}}}}{{\partial_{k_{y}}\beta}_{\eta}\left( k_{x},k_{y} \right)_{k_{y}=k_{\mathrm{inc}}}}\left\vert t \right\vert^{2}=\frac{\sin(k_{y,\mathrm{tran}}a_{y})}{\sin(k_{y,\mathrm{inc}}a_{y})}\left\vert t \right\vert^{2},$ | **(S7)** |
| --- | --- |

where $k_{y,\mathrm{tran}}=0$ for TIR, such that $R+T=1$ holds for TIR as well.

1. Higher Floquet orders and Bloch periodicity:

Typically, one expects the band structure of a periodic system with unit vector $\vec{a_{1}}=a_{x}\hat{x}$ in real space to be periodic in momentum space (reciprocal space) with a period of $\frac{2\pi}{a_{x}}$. An array of tilted waveguides does not follow this rule, because the unit vectors are not orthogonal. The other unit vectors of an array of tilted waveguides are $\vec{a_{2}}=a_{y}\hat{y}$ and $\vec{a_{3}}=\frac{a_{x}}{2}\left( \hat{x}+\frac{1}{\eta}\hat{z} \right)$; see Fig. S2(a). These yield the following reciprocal lattice unit vectors: $\vec{b_{1}}=\frac{2\pi}{a_{x}}\left( \hat{x}-\eta\hat{z} \right)$, $\vec{b_{2}}=\frac{2\pi}{a_{y}}\hat{y}$ and $\vec{b_{3}}=\frac{4\pi\eta}{a_{x}}\hat{z}$ (see Fig S2(b)). For simplicity, one can put aside the periodicity in *y*, and consider only the other two units vectors. The periodicity in *z* allows the band structure to exhibit higher Floquet orders in $\beta$, such that equation (5) in the main text needs to be amended by:

|  | $\beta_{\eta}\left( k_{x},k_{y,\mathrm{inc}} \right)=\beta_{-\eta}\left( k_{x},k_{y,\mathrm{tran}} \right)+\frac{4\pi\eta}{a_{x}}m$ | **(S8)** |
| --- | --- | --- |

where $m$ is an integer^7^. The case of $m\neq0$ describes coupling between different Floquet orders. However, this effect does not exist under our parameter settings, as it requires stronger coupling in $y$ between adjacent waveguides or shallower $\eta$ (see also the supplementary information in Ref. ^7^). We therefore restrict ourselves to the case of $m=0$ in the main text.


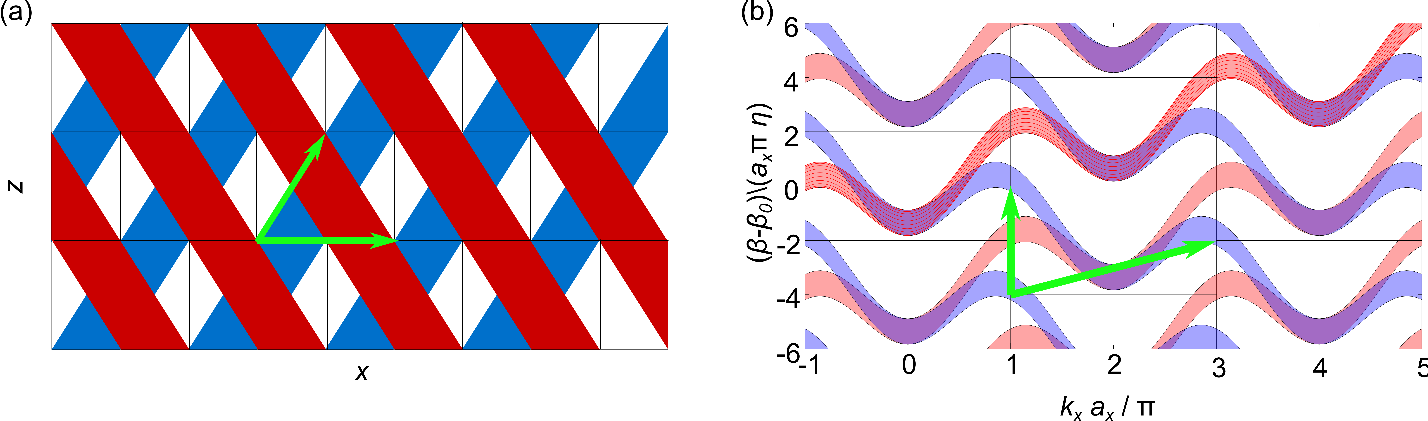


**Figure S2: Unit vectors and dispersion relations for the upper (red) and lower (blue) arrays**. a) Top-view sketch of the lattice with unit vectors in real space. b) Projection of $\beta$ as a function of $k_{x}$ with Floquet-Bloch replicas and reciprocal unit vectors. The potential describing the crossing 1D arrays tilted at opposite angles is periodic in *z*, hence the Floquet effect replicates the bands. Note that the dispersion relation has a period which differs from the conventional $\frac{2\pi}{a_{x}}$ in $k_{x}$. In principle, Floquet coupling can occur if the red band intersects with a blue band of next higher (or lower) $\beta$. However, our parameters are chosen so as to avoid Floquet coupling.

**References**

1. Hohmann, J. K., Renner, M., Waller, E. H. & von Freymann, G. Three-Dimensional μ-Printing: An Enabling Technology. *Adv. Opt. Mater.* **3**, 1488–1507 (2015).

2. Dottermusch, S., Busko, D., Langenhorst, M., Paetzold, U. W. & Richards, B. S. Exposure-dependent refractive index of Nanoscribe IP-Dip photoresist layers. *Opt. Lett.* **44**, 29–32 (2019).

3. Jörg, C., Letscher, F., Fleischhauer, M. & von Freymann, G. Dynamic defects in photonic Floquet topological insulators. *New J Phys* **19**, 083003 (2017).

4. Jörg, C. Interfaces and defects in topological model systems of 3D micro-printed waveguides. (TU Kaiserslautern, 2019). <https://kluedo.ub.uni-kl.de/frontdoor/index/index/docId/5764>

5. Szameit, A. *et al.* Fresnel’s laws in discrete optical media. *New J. Phys.* **10**, 103020 (2008).

6. Plotnik, Y. *et al.* Analogue of Rashba pseudo-spin-orbit coupling in photonic lattices by gauge field engineering. *Phys Rev B* **94**, 020301 (2016).

7. Lumer, Y. *et al.* Light guiding by artificial gauge fields. *Nat. Photonics* **13**, 339–345 (2019).
